# Moisture Adsorption potentials and energy models of *Gongronema latifolium* leaves dried in separate environments

**DOI:** 10.1002/fsn3.2616

**Published:** 2021-11-10

**Authors:** Onyekwere Ojike, Eunice Osinachi Uzodinma, Euphemia Ogochukwu Ali, Blessing C. Nweze, Chigozie F. Okoyeuzu, Chinwendu R. Eze

**Affiliations:** ^1^ Department of Agricultural and Bioresources Engineering/ African Centre of Excellence for Sustainable Power and Energy Development University of Nigeria Nsukka Nigeria; ^2^ Department of Food Science and Technology University of Nigeria Nsukka Nigeria

**Keywords:** energy models, leaf grits, moisture adsorption isotherms, moisture content, water activity

## Abstract

Moisture adsorption isotherm potentials and energy models of *Gongronema latifolium* leaf grits were investigated. Fresh leaves were dried in sun; passive solar dryer and hot air oven, while proximate composition of the dried and fresh leaves were determined using standard laboratory procedure. Equilibrium moisture content (EMC) of the leaf grits was measured using gravimetric static method. Selected mathematical and statistical models were applied on the experimental data to evaluate data fitting. Energy calculations were done based on the mathematical models. The EMCs of the leaf grits directly increased with water activity (a_W_) at specific temperatures. Adsorption data was better represented by GAB model than others while differential enthalpy decreased as the EMC of the oven sample increased. Differential entropy of all the samples decreased as the EMC increased. The safest monolayer moisture content varied between 7.1036 and 8.0164 gH_2_O/100 g solid, below 40°C, within relative humidity of 10%–50%. Sun and oven leaf grits adsorbed more moisture than solar sample. Proximate contents of the dried leaves showed higher values for protein and ash. Overall results indicated that the leaf grits when properly packaged could be used as spice or tea powder to manage household nutrient security in addition to the use as therapeutic foods.

## Graphical abstract

Moisture adsorption isotherm potentials for the culinary and medicinal leafy vegetable, Gongronema latifolium leaf was determined. This was to use it round the seasons of the year to maintain healthy lifestyle and spoilage/post‐harvest loss. Study gave room for making choice of the critical moisture content, storage temperature and relative humidity/water activity range in which the dried leaves can be packaged and stored for regular consumption.

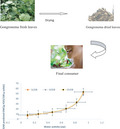

## 1 INTRODUCTION

Herbaceous vegetables have unique benefits within the farming systems and household gardens because they grow quickly. Many are disease‐ and drought‐resistant since they easily adapt to local and environmental conditions. They are counted as part of the most inexpensive sources of different kinds of nutrients, sustainable and culturally acceptable. These vegetables can also fit well into daily dieting and could serve as a pathway to handling nutrient‐hunger found in most households in third world countries and beyond, in addition to general maintenance of human health. As culinary herbs and spices in which dried or fresh leaves are used for cooking (Ogunlesi et al., 2010), can enhance the flavor and taste of foods and as a source of dietary medicine as noted by Uhegbu et al. (2011).

*Gongronema latifolium* (“Utazi”) leaves, among others, are broadly found in Africa, South America, and other tropical and subtropical regions as indicated by Offor (2014). Different parts of the plant are used for different purposes. The leaves are used primarily for culinary and medicinal purposes. Some pharmacological studies indicated that the leaves have analgesic, antimicrobial, antibacterial, anti‐ulcer, antisickling, antioxidant, antiasthmatic, antipyretic, hypoglycemic, and anti‐inflammatory properties (Okpala, 2015). Reports of Edet et al. (2011) showed that they could be used for treatment of health problems such as malaria, nausea, anorexia, diabetes, hypertension, constipation, cough, intestinal worms, dysentery, and dyspepsia including microbial infections (viral hepatitis, bilharzias, among others).

The main method of preservation of the leaves is drying due to its perishability. However, proper packaging and storage of the dried leaves is still a problem. During the drying process, moisture is simultaneously transferred out of the fresh food in the presence of heat until low moisture content is obtained for storage and safety of the food product. This is done to reduce water activity of the food preventing free water from encouraging biochemical reactions that lead to spoilage by microorganisms. Water activity (a_w_) of a food equals the relative humidity above it divided by 100. Equilibrium relative humidity (ERH) becomes equal to water activity after multiplying a_w_ with 100%, indicating a point of saturation. The equilibrium moisture content (EMC) for a food substance at a particular environment connotes the moisture content the food will attain when kept in that environment for an indefinite period. However, EMC at drying process represents the moisture content attained by the food material when it can no longer gain or lose water and is at vapor equilibrium with its surrounding. Hence, all the free water would have been removed. Dincer and Esin (1995) noted that the ERH of air in contact with the food substance decides if it will gain or lose moisture in a given surrounding.

Consequently, adsorption isotherm acts as an instrument for making proper choice of packaging material and storage atmosphere that will ensure retention of aroma, taste, flavor, appearance, texture, nutrients, and mixing of powdered food products into food matrix during food preparations. It also helps to limit microbial deterioration in hygroscopic and other foods. Adsorption isotherm studies involve temperature and energy. As a result, net isosteric heat of sorption, differential entropy, and Gibb's free energy changes among others, which define thermodynamic properties, should be calculated based on mathematical models describing the experimental adsorption data. Differential enthalpy helps one to discover variations in energy content when water molecules mix with the hygroscopic food during adsorption process that is, water–food material interaction. Entropy changes assists in the estimation of interaction resulting from binding or repulsive forces taking place within the adsorption system, while Gibb's free energy change explains how spontaneous the adsorption process was.

Researchers such as Pedro et al. (2010) noted that adsorption potential of a food product could be affected by drying methods applied, as changes such as solubility, shrinkage, rehydration, shape, size, porosity, and density; can occur. Energy calculations depending on sorption models generally help in design and optimization of dehydration equipment, making proper choice for storage atmosphere, and packaging materials for the food products (Kaymak‐Ertekin & Gedik, 2004). Research works on adsorption studies for agro‐products as indicated by Pedro et al. (2010), among others, are numerous but information on *Gongronema latifolium* leaves is very scarce. Therefore, the goal of the research work was to estimate monolayer moisture content and best temperature range in which the dried leaves can be stored; calculate values for thermodynamic parameters based on the statistical and mathematical models, for proper choice of drying environment for the fresh leaves including designing of suitable dehydration equipment. Adsorption studies were carried out at 30, 40, and 50°C in which the leafy vegetable could be exposed during storage period.

## 2 MATERIALS AND METHODS

### 2.1 Materials and sample preparation

*Gongronema latifolium* fresh leaves were purchased from “Ogige” market in Nsukka town of the University. The leaves were identified in Department of Botany, University of Nigeria, Nsukka (UNN). Solar dryer, liquid‐in‐glass thermometer (110°C), digital weighing balance (Ohaus‐3 kg, England), stainless steel bowls, plastic sieves, and distilled water were used. The materials were obtained from National Centre for Energy Research and Development (NCERD), UNN, biomass laboratory.

**FIGURE 1:**
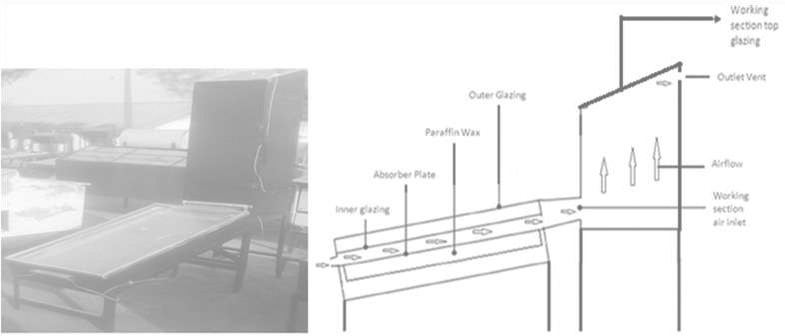
Passive solar dryer used for the study

The leaf twigs were destalked, washed with clean water, and drained. A 600 g of the leafy vegetable was weighed and subjected to sun, solar, and oven drying. Prior to the drying, initial moisture content of the fresh leaves was determined. The quantity of leaves dried was to be sure of having enough dried leaves for entire study. A passive solar dryer fabricated by the NCERD (Figure 1), UNN, and LAB AIDS, −1201‐Indian hot air oven from the Microbiology laboratory of the department of Food Science and Technology, UNN, were used, respectively, for solar and oven drying of the leaves. During drying, the ambient and drying chamber temperatures were monitored. The leaves were evenly spread during the drying operations to ensure effective drying and were weighed at intervals of two hours until a constant weight was obtained. Drying was carried out in the middle of March, 2018 and the duration for open air/sun and solar drying were 6 and 5 days, respectively. Oven drying took approximately 4 days at 55°C. The period of mid‐month of March was chosen for the study because it corresponds with the time of the effect of equinox in Nsukka (latitude 6.8^0^) due to its closeness to the equator (Ojike, 2011; Uzodinma et al., 2020). Equinox leads to clear sky solar radiation which is usually at its peak, between the hours of 10 a.m. and 4 p.m. Hence, the leaves for the open/air sun drying were usually sprayed for drying by 10 a.m. and at 4 p.m. they were properly wrapped with polythene to hinder moisture loss and taken to a shade. Drying during equinox and wrapping with polythene at the end each drying section ensures relatively constant temperature and reduces the environmental impact on the drying. For the solar dryer, day and night average temperatures were recorded as: day time‐ambient temperature equals 33°C; glazing temperature equals 57°C; absorber plate temperature equals 72°C; heat storage temperature equals 48°C and drying chamber temperature equals 68°C. Night time temperatures varied between 25 and 29°C for all the parameters. The average drying chamber relative humidity was 45.4%, while the ambient relative humidity average was 64.7%. Wind speed at the ambient surroundings of the dryer was recorded as 1.79 ms^−1^ on average. After drying, the leaves were separately ground into coarse powder (2.778 mm size), packaged in polyethylene bags and kept in airtight containers inside food processing laboratory as SUDB‐sun‐dried sample; SODB‐solar‐dried sample; and OVDB‐ oven‐dried sample; for further studies.

### 2.2 Proximate analyses of fresh and dried leaves

Proximate parameters (percentage moisture, crude protein, crude fat, crude fiber, ash, and carbohydrate by difference contents) of the fresh and dried leaves were determined using AOAC (2010) method.

#### 2.2.1 Moisture adsorption studies of the leaf grits

The gravimetric method of sorption study described in Labuza (1984) was used to measure the EMC of the powdered leaves at 30, 40, and 50°C. An incubator was used as temperature control chamber. The experimental setup consists of twelve airtight plastic containers (1,000 ml each) containing different concentrations of sulfuric acid (H_2_SO_4_) solutions (15, 25, 35, 45, 55, and 65%), corresponding to six levels of water activity (a*_w_*) between 0.0972 and 0.9811 (Ruegg, 1980). The different concentrations of H_2_SO_4_ were previously prepared using density formula (Equation 1):

(1) $$\rho =\frac{m}{v}$$

Where *ρ* is the density of the acid, m is the mass of acid, and v is the volume of the acid.

The sulfuric acid solutions were allowed to cool at room temperature before transferring 600 ml each into the containers. Duplicate samples (0.5 g each) of dried samples were weighed into cleaned crown corks with known weights. The corks were placed in the containers and were supported with wire gauze fixed above the solution to prevent them from falling. The containers were then tightly closed and placed in an incubator set at selected temperatures (30, 40, and 50°C) for the samples to attain EMC. Small glass bottle containing toluene (1.5 ml) was placed inside the container to suppress microbial deterioration at high relative humidity. The crown corks were weighed after every 48 h until the difference between two consecutive weights were less than 0.5% of the initial weight of the sample. The final moisture content of the samples which is the EMC was determined by material balance from the initial moisture content using equation 2 from the initial moisture content for dried samples meant for adsorption. The total time for removal, weighing, and putting back the samples in the containers ranged from 2 to 5 min as recommended by Cooperative Project Cost 90 (Gal, 1988). This helped to limit the degree of atmospheric moisture sorption during weighing.

(2) $$\frac{M}{100\left(W_{1}\right)}+\left(W_{3}‐W_{2}\right)=\frac{\text{EMC}}{100}\times \left[W_{1}+\left(W_{3}‐W_{2}\right)\right]$$

where M is the initial moisture content of the sample, W_1_ is the weight of sample used during adsorption, W_2_ initial weight of sample and crown cork, W_3_ is the final weight of sample and crown cork at equilibrium, and EMC is the equilibrium moisture content.

### 2.3 Moisture Adsorption models and fitting the models

EMCs, water activities, and temperature data were fitted into four sorption kinetic models of BET‐Brunauer‐Emmet‐Teller, GAB‐Guggenheim‐Anderson‐de Boer, Oswin, and Henderson; as shown by Equations (3), (4), (5), (6), (7) (McKenna, 1984). The models were chosen based on versatility, relatively simple mathematical computation, and broad usage in modeling in experimental sorption data of many foods particularly fruits and vegetables. The methods can also describe sorption isotherms of agro‐foods within the water activity range of 0.00–0.95 as noted by Iguedjtal et al. (2008). The parameters of the models were estimated from the experimental results using the nonlinear regression function of SPSS version 23.0 (SPSS Inc.). Adequacy of each model used was evaluated using three statistical models: coefficient of determination (r^2^), for the goodness of fit for each model, the root mean square error (RMSE %) between the experimental and predicted EMCs which determines the accuracy of fit of the models and the reduced Chi square (χ^2^) which shows the suitability of the models. These parameters were calculated using the Equations 8, 9, 10, and 11, 12, 13 as found in Lomauro et al. (1985).

(3) $$\text{BET;}\;\text{EMC}=\frac{M_{o}Ca_{w}}{\left[\left(1‐a_{w}\right)+\left(C‐1\right)\left(1‐a_{w}\right)a_{w}\right]}$$

(4) $$\text{GAB;}\;\text{EMC}=\frac{M_{0}CKa_{w}}{\left[\left(1‐Ka_{w}\right)\left(1‐Ka_{w}+CKa_{w}\right)\right]}$$

(5) $$\text{GABC;}\;C=K\exp\left(\frac{\text{Hm}‐\text{Hn}}{RT}\right)$$

(6) $$\text{Oswin;}\;\text{EMC}=C{\left(\frac{a_{w}}{1‐a_{w}}\right)}^{A}$$

(7) $$\text{Henderson};\;\text{EMC}={\left\{\frac{‐\ln\left(1‐a_{W}\right)}{C}\right\}}^{\frac{1}{A}}$$

where MC is the observed or predicted moisture content (% dry basis), M_O_ is the monolayer moisture content (g/g dry solid), a_W_ is the water activity (decimal); A, C, and K are the moisture sorption constants (dimensionless); Hm = total heat of sorption of the first layer (kJ.mol^−1^), Hn = total heat of sorption of the multilayer (kJ.mol^−1^); R = universal gas constant, T = temperature

(8) $$r^{2}=\pm \frac{\text{explained}\;\text{variation}}{\text{total}\;\text{variation}}=\pm \frac{\sum {\left(\text{Yest}‐y_{\text{mean}}\right)}^{2}}{\sum {\left(Y‐y_{\text{mean}}\right)}^{2}}$$

(9) $$r^{2}=1‐\frac{\text{ESS}}{\text{TSS}}$$

(10) $$r=\pm \sqrt{r^{2}}$$

where r = coefficient of correlation; r^2^ represents coefficient of determination for goodness of fit

(11) $$\text{RMSE}=\sqrt{\frac{1}{N}\left[\sum_{i=1}^{N}{\left({\frac{\sum_{i=1}^{N}\left({\text{EMC}}_{\exp,i}‐{\text{EMC}}_{\text{pred},i}\right)}{N‐1}}^{2}\right)}^{2}\right]}$$

(12) $$X^{2}=\frac{\sum_{i=1}^{N}{\left({\text{EMC}}_{\text{exp,}i}‐{\text{EMC}}_{\text{pred},i}\right)}^{2}}{N‐1}$$

The average percentage difference between the experimental and predicted values, P, was calculated thus (Equation 13):

(13) $$P\left(\%\right)=\frac{100}{n}\sum_{i=1}^{n}\frac{\left({\text{EMC}}_{\exp,i}‐{\text{EMC}}_{\text{pred},i}\right)}{{\text{EMC}}_{\exp,i}}$$

where ESS and TSS are the error and total sum of squares, respectively. EMC_exp,i_ is the ith value of the experimentally measured EMC, EMC_pred,i_ is the ith predicted value of the EMC, N is the number of observations.

The P is the average percentage difference (the relative deviation) and n is the number of constants. A model was considered good, accurate, and suitable when the value of r^2^ is high (close to 1), the value of P is less than 10% (Lomauro et al., 1985) and both RMSE and reduced Chi square values are low.

#### 2.3.1 Energy models/thermodynamics properties

Thermodynamic properties of the moisture adsorption isotherms of the dried leaves such as apparent area of adsorption, enthalpy or net isosteric heat of adsorption, and differential entropy were evaluated.

##### Determination of isosteric heat of adsorption

The concept of isosteric heat of sorption or enthalpy of sorption (q_st_) which shows the effect of temperature on the isotherm was determined by equation 14 derived from the Clausius‐Clapeyron Equation 15 (Argyropoulos et al., 2012).

(14) $$q_{\text{st}}=q_{t}‐△H_{\text{vap}}$$

The net isosteric heat of sorption (q_st_) is thermodynamically given by Equation 15:

(15) $$q_{\text{st}}=\frac{RT_{1T_{2}}}{T_{2}‐T_{1}}\ln\frac{aw_{2}}{aw_{1}}$$

where q_st_ is the net isosteric heat of sorption (enthalpy of sorption), q_t_ is the total heat of sorption, ΔH_vap_ is the heat of vaporization (kJ/mol) of pure water, R is the universal gas constant (8.314 J mol^−1^ K^−1^), a_w2_ and a_w1_ are the water activity values at absolute temperatures T_2_ and T_1_ (K), respectively.

##### Determination of adsorption entropy change

The sorption entropy (differential entropy) (ΔS^o^) which interprets processes such as dissolution, crystallization, and swelling of the samples was determined as follows (Equation 16):

(16) $$‐\ln\left(a_{w}\right)=\left(\frac{q_{\text{st}}}{R}\right)\frac{1}{T}‐\left(\frac{△S^{o}}{R}\right)$$

where a_W_ is water activity, $q_{\mathrm{st}}$ is net isosteric heat of sorption, R is the universal gas constant (8.314 J mol^−1^ K^−1^), T is temperature, and $△S^{o}$ is differential entropy.

##### Apparent area of adsorption (S_o_)

The monolayer moisture values from BET model were used to evaluate the apparent area of adsorption of the sorbent using the following Equation 17:

(17) $$S_{o}=\frac{1}{M_{s}}N_{o}AM_{o}$$

where N_o_ is the Avogadro's number (6.023 × 10^23^ molecules/mole), A is the apparent surface area of one water molecule (1.05 × 10^−19^m^2^), M_s_ is the molar mass of water (18 g/mol), M_o_ is the monolayer moisture content (gH_2_O/g solid), and S_o_ is the apparent area of sorption (m^2^ /g solid).

#### 2.3.2 Monolayer moisture content dependency on the storage temperatures

Effect of temperature on the moisture adsorption isotherm was considered by relating monolayer moisture content with temperature (Hector et al., 1986). This was based on Arrhenius chemical kinetic model‐M_k_ = M_o_exp(‐E_a_/RT) (Rao, 1999). ln (M_k_) was plotted against 1/T where M_k_ represents critical moisture contents of the samples during moisture adsorption isotherm process; M_ok_ = frequency factor; E_a_ = activation energy; R‐universal gas constant (J/mol/K); T‐temperature in Kelvin (K).

#### 2.3.3 Statistical analysis

Experimental design for the proximate composition of fresh and dried leaves was based on completely randomized design. All the data obtained were analyzed using one‐way analysis of variance. Mean separation was done using Duncan's new multiple range test and the level of significance was accepted at *p* < .05. Sorption and thermodynamic properties of the leaf grits were designed as explained in sections 2.3–2.3.2

## 3 RESULTS AND DISCUSSION

### 3.1 Weight loss of the leaves under different drying methods

**FIGURE 2:**
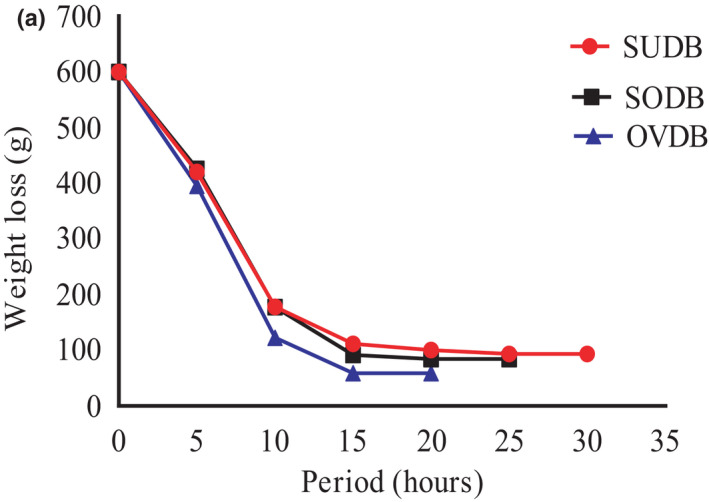
Weight loss curve for *Gongronema* leaves. SUDB, sun dried *Gongronema* leaves, SODB, solar dried *Gongronema* leaves and OVDB, oven dried *Gongronema* leaves

The pattern for weight loss of *Gongronema* leaves is shown in Figure 2. Ambient temperature at the period of drying ranged from 22.5 to 33.5°C. Sun and solar drying of the fresh leaves took 30 and 25 h (5 h/day) of the drying period, respectively, while oven drying lasted for 20 h (5 h/day). Oven samples dried within approximately within 4 days at the constant temperature (55°C); solar samples dried within 5 days while open air/ sun samples dried within 6 days. Initial moisture content of the fresh leaves was 75.54%. Results indicated that oven drying method yielded higher weight loss (540.8 g) for the leaves than the sun and solar drying methods. A moisture loss for sun drying was 506.1 g, while solar drying resulted to 516. g. The higher weight loss from the oven‐dried samples could be due to heat supply from the oven at relatively constant temperature (55°C) than in sun and solar drying processes.

Weight loss curve indicated that initially water was removed by evaporation from the saturated surface of the fresh leaves before the area of the surface gradually decreased followed by water evaporation from interior portion of the leaves that determined the drying rate. The drying was continued until critical moisture content was reached before the falling rate period. The drying rate continued to decrease linearly and EMC was attained where the weight loss for samples remained constant. At this point, no water was lost or gained by the samples and constant weights were repeatedly obtained before exiting the process.

### 3.2 Proximate composition of the dried and fresh samples

Proximate composition of fresh and dried *Gongronema latifolium* leaves are presented in Table 1. Mean moisture content of the fresh leaves was 75.54%. Variations might occur in moisture composition of leafy vegetables due to the time and season of harvest. Those harvested during rainy season contain high moisture due to high relative humidity and low temperature than those harvested during dry season. The moisture content of food gives an indication of its shelf‐life and nutritive values and low moisture content is required for long storage life. Morris et al. (2004)’s findings indicated that removal of moisture leads to an increase in concentration of nutrients in the plant produce. Consequently, higher values of protein were recorded for the dried samples than for fresh leaves with solar‐dried sample having highest value. This could imply that drying of the fresh leaves under solar dryer environment has greater advantage in concentrating crude proteins and other nutrients in the leaves. This highly recommends the dried leaves as cheap and readily available alternative protein source as shown in Fredrick et al. (2008) and Okoli et al. (2019). Fresh samples dried in solar dryer environment also had significantly (*p* < .05) higher values in ash and crude fat than other samples. However, there was no significant (*p* > .05) difference in the crude fiber content of solar and oven‐dried samples. The carbohydrate contents of all the leaves were low. However, the fresh leaves had significantly (*p* > .05) lower value than others. This could show that leafy vegetables contribute slightly to energy content when consumed except in adjunct with other foods as indicated by Rosello et al. (2000). Also, Uwaegbute (1989) pointed out that vegetables were not rich sources of dietary energy and might be due to high moisture and crude fiber contents in addition to low fat composition.

**TABLE 1:** Proximate composition of the dried leaves and parameters of Arrhenius‐type equation for the effect of temperature on monolayer moisture content of *Gongronema* leaf grits

Samples	Moisture (%)	Crude protein (%)	Crude fat (%)	Crude fiber (%)	Ash (%)	Carbohydrate by difference (%)
FRB	75.54^d^ ± 0.06	12.82^a^ ± 0.06	2.78^a^ ± 0.04	4.38^a^ ± 0.03	1.98^a^ ± 0.04	2.52^a^ ± 0.22
SUDB	7.01^c^ ± 0.03	48.35^b^ ± 0.07	3.99^b^ ± 0.04	6.77^b^ ± 0.04	8.25^b^ ± 0.04	25.63^d^ ± 0.21
SODB	6.83^b^ ± 0.05	54.22^d^ ± 0.06	5.35^d^ ± 0.01	7.81^d^ ± 0.01	10.28^d^ ± 0.04	15.51^b^ ± 0.09
OVDB	6.02^a^ ± 0.01	51.98^c^ ± 0.07	4.78^c^ ± 0.06	7.59^c^ ± 0.03	8.99^c^ ± 0.01	20.64^c^ ± 0.13

	ln (M_ok_)	E_a_ (kJmol^−1^)	r^2^	Sy.x	Trend line
SUDB/SUDA	1.26	3.74	0.32	0.09390	Y = −450.0X + 3.507
SODB/SODA	0.92	4.16	0.68	0.04899	Y = 500.0X + 0.40
OVDB/OVDA	1.71	8.73	0.79	0.07757	Y = −1050X + 5.503

Note

Values on the table are means of triplicate determinations ± standard deviation. Means with the same superscript on the same column are not significantly different at *p* > .05.

Abbreviations: E_a_, activation energy; M_o_, frequency factorOVDB, oven dried Gongronema leaf powder; SODB, solar dried *Gongronema* leaf powder; SUDB, sun dried Gongronema leaf powder.

### 3.3 Moisture adsorption isotherm potentials of *Gongronema latifolium* leaf grits

**FIGURE 3:**
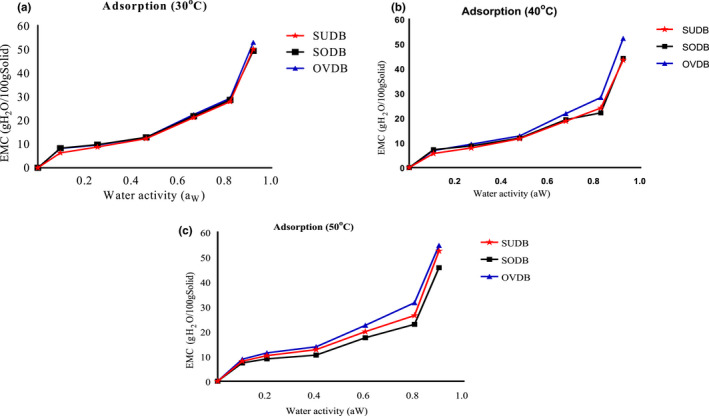
(a) Equilibrium moisture content (db) versus water activity for *Gongronema* leaf grits at 30oC. (b) Equilibrium moisture content (db) versus water activity for *Gongronema* leaf grits at 40oC. SUDB, sun *Gongronema* leaf grits, SODB, solar *Gongronema* leaf grits and OVDB, oven *Gongronema* leaf grits. (c) Equilibrium moisture content (db) versus water activity for *Gongronema* leaf grits at 50oC

**FIGURE 4:**
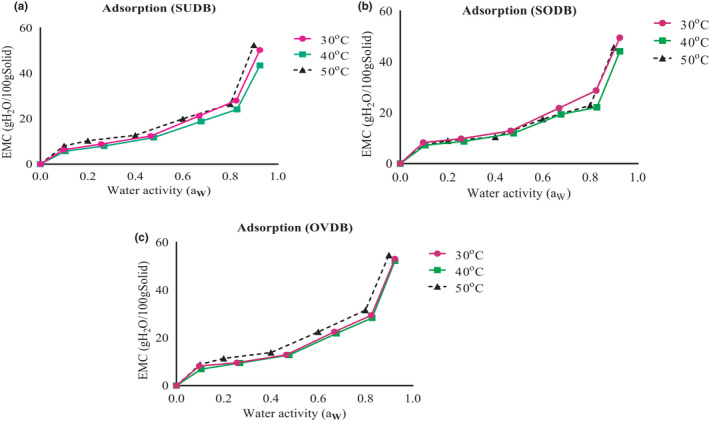
(a) Equilibrium moisture content (db) versus water activity for Sun‐ *Gongronema* leaf grits at different temperatures. (b) Equilibrium moisture content (db) versus water activity for Solar‐ *Gongronema* leaf grits at different temperatures. (c) Equilibrium moisture content (db) versus water activity for Oven‐ *Gongronema* leaf grits at different temperatures. SUDB, sun *Gongronema* leaf grits, SODB, solar *Gongronema* leaf grits and OVDB. oven *Gongronema* leaf grits

**FIGURE 5:**
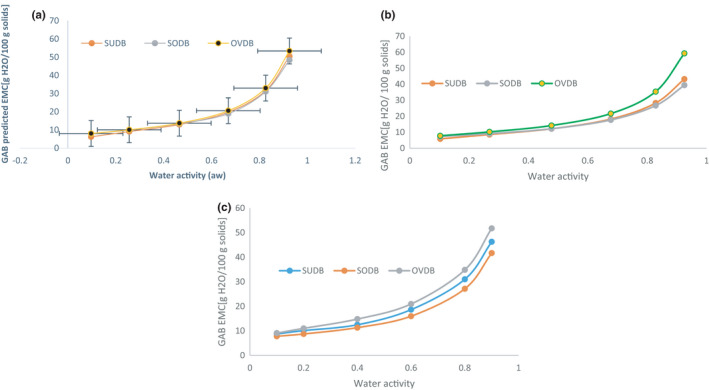
(a) GAB predicted EMC versus water activity of the *Gongronema* leaf grits at 30OC. (b) GAB predicted EMC versus water activity of the *Gongronema* leaf grits at 40 OC. (c) GAB predicted EMC versus water activity of the *Gongronema* leaf grits at 50 OC. SUDB, sun *Gongronema* leaf grits, SODB, solar *Gongronema* leaf grits and OVDB, oven *Gongronema* leaf grits

Experimental data for the EMC of sun, solar, and oven *Gongronema* leaf grits corresponding to various water activities (a_W_) for three different storage temperatures (30°C, 40°C, and 50°C) are shown in Figures 3 and 4. The GAB predicted EMC versus water activity for the different temperatures is shown in Figure 5. The EMC was calculated using equation 4 for each water activity and temperature level. Sun and oven‐dried leaves adsorb more moisture than the solar‐dried leaves. This might be due to differences in pore size and binding site. Thermal effect from the oven may have caused the exposure of more water‐binding sites in the matrix. The EMC of the leaf powder directly increased with a_W_ at selected temperatures not withstanding the drying method used. This could be due to increase in water vapor pressure present in food with that of the surrounding and agreed with the findings of Shivhare et al. (2004) in adsorption study using mushroom. The trend is common to all food materials and it is an indication that the leaves would adsorb more water at higher relative humidity/water activity. The Figures also showed that at water activity (a_W_) greater than 0.5, a higher amount of water was adsorbed for a small rise in a_W_. This is because at low water activities physical sorption occurs on strongly active binding sites of substrate, such as –OH groups present on the surface film. In the intermediate a_w_ range, adsorption took place at less active sites and showed that the region could be a zone highly unstable in terms of spoilage.

Similar behavior was reported by other authors for different hygroscopic foods (Ariahu et al., 2006). All the moisture adsorption isotherm curves typically displayed a sigmoid shape corresponding to type II isotherms (Mathlouthi & Rogé, 2003) that was dependent on Bruaneur et al. (1940) classification of moisture sorption isotherms. The type II isotherm is known for the formation of multiple layers of adsorbate molecules at the internal food solid surface and is a characteristic specific to most organic tissues and commonly observed in hygroscopic food products (Rodriguez‐Bernal et al., 2015).

#### 3.3.1 Influence of temperature on the adsorption potentials of the leaf grits

**FIGURE 6:**
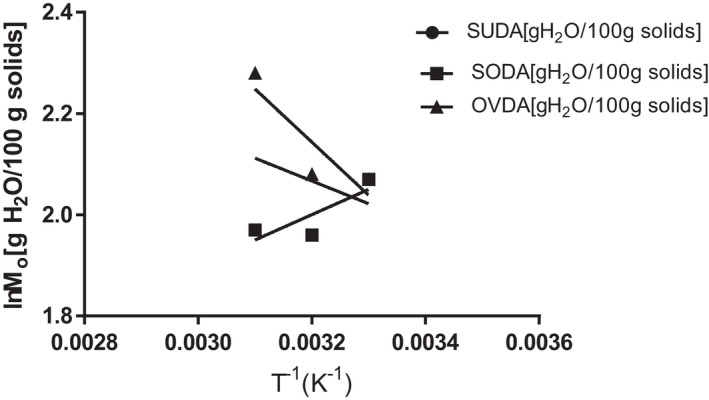
Relationship between Monolayer moisture of leaf grits and Temperature (K‐1) based on Arrhenius kinetic model. SUDA, sun *Gongronema* leaf grits, SODA, solar *Gongronema* leaf grits and OVDA, oven *Gongronema* leaf grits; 30oC=0.0033/K, 40oC=0.0032/K, 50oC=0.0031/K

The effect of temperature is displayed by Figures 3 and 4. Influence of temperature on adsorption isotherm is highly remarkable because during processing and storage, foods are exposed to various temperatures; and water activity varies with temperature. This change in water activity concerning temperature variation has shown to be directly related to the rate of food spoilages notably reported by Iglesias and Chirife (1982); Labuza (1984). Hence, for most dry foods, an increase in water activity of 0.1 may decrease the shelf‐life by a factor of two to three folds. Overlapping of values for the different temperatures was observed for sun‐dried sample and others, during adsorption process but its moisture adsorption was less at 40°C (Figure 3b) than in 30 and 50°C. For solar dried sample (Figure 4b), moisture adsorption was higher at 30°C than at 40 and 50°C. In the case of oven‐dried sample, adsorption was high at all temperatures (Figure 4c) than for sun and solar samples. This might have resulted from the effect of different drying environments on the leaves. There was no significant (*p* > .05) difference in moisture adsorption at 30°C (Figures 5a and 6). Some researchers also noted that the composition of some dehydrated foods such as fiber, carbohydrates, and other smaller components could be a hindrance to moisture adsorption (Yogendrarajah et al., 2015). Research reports of Palipane and Driscoll (1992) showed that increase in temperature causes activation of the water molecules raising them to higher energy levels. This would have allowed the molecules to break away from their adsorption sites giving rise to decrease in EMC, especially for the oven sample.

#### 3.3.2 Monolayer moisture content of the leaf grits versus temperature based on chemical kinetics

This is shown in Figure 6. The sun and oven monolayer moisture contents were above 2.0 at both 40 (0.0032 K^−1^) and 50°C (0.0031 K^−1^). However, at 30°C (0.0033 K^−1^) there were no significant (*p* > .05) differences in monolayer moisture contents of the samples from the separate environments. The reason could be obvious based on the kinetic model. Increasing temperature or decreasing the energy of activation (E_a_) of particles of reactants will lead to increase in the rate of moisture adsorption and vice versa. At 30°C which was the lowest storage temperature, the level of excitation of the molecules of the reactants could be similar and implied that the number of collisions per second that might lead to moisture adsorption or not, behaved similarly. Hence, the food products from separate drying environments adsorbed moisture without significant (*p* > .05) differences. As the temperature increased to 50°C, the rate of moisture adsorption by the products differed. Table 1 shows results of the Ea, M_o_, among others. The minimum energy required for monolayer moisture contents adsorption and Mo_k_ values were higher for sun and oven‐dried products than solar sample. This might imply more of the grit particles binding with water than for the solar sample as the temperature increased. Sun‐dried sample had the poorest r^2^ among others and might be due to daily changes in the weather conditions (Uzodinma et al., 2014). However, the scenario might suggest: (i) the need to further carryout studies to find out what caused less moisture adsorption by the solar dryer sample for proper understanding and guidance on design and construction of the drying equipment; (ii) Packaging of the samples for storage and distribution should have slight modifications for each to have longer shelf life.

#### 3.3.3 Moisture adsorption models and their adequacies

Evaluation of the models as shown by Equations (3), (4), (5), (6), (7), (8), (9), (10), (11), (12), (13), (14), (15), (16), (17) and the suitability for fitting experimental data are presented in Table 2. Monolayer moisture content (M_o_) of the sun, solar and oven *Gongronema* leaf powder from GAB model ranged from 7.1036 to 9.7456 gH_2_O/100g solid. The M_o_ is the moisture content at which the rate of biochemical, enzymatic, and microbial reactions will negligibly occur due to strong binding of water to the surface (Yogendrarajah et al., 2015). Hence, it is an important parameter for choosing suitable storage conditions for hygroscopic food products. The M_o_ values of the leaves relatively showed an inverse variation with the temperature from 30 to 40°C, but increased at 50°C despite any of the drying method applied. This might be due to decrease in number of active sites for water binding because of the physical and chemical changes caused by temperature. Also, increase in temperature could activate the water molecules of the food due to increase in energy levels, causing less stability and break away from the water‐binding sites. Thus, the monolayer moisture content of the food decreased and agreed with the reports of Ariahu et al. (2006) who used tropical water crayfish for adsorption studies, among other authors. However, increase in M_o_ with the temperature might be due to more opening of new binding sites or some of its components becoming increasingly hydrophilic as the temperature increases. This can allow greater water vapor molecules to bind, resulting to increase in M_o_.

**TABLE 2:** Estimated adsorption model constants, monolayer moisture content‐M_o_ (gH_2_O/100 g dry solid), and statistical parameters for *Gongronema* leaf grits

Model	Sun drying			Solar drying			Oven drying		
	30	40	(50°C)	30	40	(50°C)	30	40	(50°C)
GAB									
M_o_	7.8655	7.3049	8.5608	7.8858	7.1036	7.1617	7.9465	8.0164	9.7456
K	0.9138	0.8996	0.9059	0.9054	0.8864	0.9202	0.9206	0.935	0.9027
C	27.827	25.7151	128.945	140.054	158.8082	762.689	136.697	66.6413	56.8337
R^2^	0.95	0.94	0.91	0.96	0.88	0.90	0.94	0.94	0.94
R	0.98	0.97	0.95	0.98	0.94	0.95	0.97	0.97	0.97
RMSE	1.6120	1.7494	3.1921	1.3684	2.7655	2.4981	1.7012	4.1380	1.9386
Χ^2^	5.1971	6.121	20.3791	3.7449	15.2962	12.4808	5.7885	34.2459	7.516
%P	5.8223	9.0168	6.5868	6.0566	4.4681	7.102	5.5084	5.8336	5.7944
r^2^	0.95	0.94	0.91	0.96	0.88	0.90	0.94	0.94	0.94
BET									
M_o_	6.2112	5.2083	7.0771	6.0606	5.102	6.7204	6.0976	5.4289	7.0922
C	53.667	48.0	471.0	55.0	65.333	186.0	164.0	230.25	135.0
R^2^	1.0	0.97	1.0	0.99	1.0	1.0	1.0	1.0	1.0
R	1.0	0.99	1.0	1.0	1.0	1.0	1.0	1.0	1.0
S_o_	2.18.2253	182.9893	248.648	212.9241	179.2545	236.157	214.2341	190.7399	249.149
RMSE	0.2779	0.1141	1.6256	0.3257	0.0779	0.5547	0.1363	0.2850	2.498
Χ^2^	0.2317	0.0391	7.9275	0.3183	0.0182	0.9231	0.0558	0.2437	18.7133
%P	2.8957	1.488	14.7461	3.0285	0.8743	5.1021	1.2186	3.1809	21.0832
r^2^	1.0	1.0	1.0	1.0	1.0	0.99	1.0	1.0	0.99
OSWIN									
A	0.442	0.436	0.404	0.387	0.383	0.398	0.405	0.434	0.405
C	15.029	13.171	17.567	16.428	14.098	15.302	16.61	15.472	19.472
R^2^	0.98	0.99	0.96	0.96	0.95	0.94	0.96	0.98	0.97
r	0.99	1.0	0.98	0.98	0.98	0.97	0.98	0.99	0.99
RMSE	2.2794	1.9389	4.4699	2.9035	3.4208	4.1350	3.3546	2.8391	3.3398
Χ^2^	7.7936	5.6389	29.9705	12.6456	17.5505	25.6472	16.8798	12.0903	16.7314
%P	8.5013	7.553	11.5083	11.0443	12.3512	13.0276	12.2209	9.2806	9.070
r^2^	0.98	0.99	0.96	0.96	0.95	0.94	0.96	0.98	0.97
HENDERSON									
A	1.499	1.513	1.627	1.646	1.642	1.621	1.569	1.498	1.657
C	0.01	0.012	0.006	0.006	0.008	0.007	0.007	0.009	0.004
R^2^	0.93	0.94	0.90	0.89	0.89	0.88	0.88	0.92	0.92
r	0.96	0.97	0.95	0.94	0.94	0.94	0.94	0.96	0.96
RMSE	4.5072	3.9117	6.1424	4.6263	4.8558	5.1271	5.0273	4.7796	4.8981
Χ^2^	30.4731	22.9519	56.5936	32.1042	35.3682	39.4306	37.9107	34.2672	35.987
%P	16.7583	15.1056	18.8375	18.223	18.0899	21.7686	21.0297	20.3069	17.5143
r^2^	0.93	0.94	1.0	0.89	0.89	0.88	0.88	0.92	0.92

Note

r^2^ = Coefficient of determination for a goodness of fit; r = Correlation coefficient/Coefficient of correlation. A, C and K are the moisture sorption constants.

The M_o_ shows the maximum amount of water that is strongly adsorbed to specific sites per gram of dry substance and a pointer to optimum value at which a food is more stable (Cassin et al., 2006; Pérez‐Alonso et al., 2006). The M_o_ values are very important since below M_o_ of a food, water is strongly bound and the rate of deteriorative reactions is minimal. Therefore, at a given temperature, the safest water activity level is that corresponding to M_o_ or less. Hossain et al. (2001) and Yogendrarajah et al. (2015) reported decrease in M_o_ with increase in temperature at different adsorption studies using pineapple and black peppercorns, respectively. The M_o_ values were also in agreement with those recorded by McKenna (1984), which indicated that the monolayer moisture content in foods was lower than 10 g/100 g dry matter. Negligible differences existed among the M_o_ values obtained for BET model at all temperatures and were less than 10 gH_2_O/100 g dry solid. Even though BET model had lower values for Mo than GAB model, the model is valid for water activity ranging from 0.1 to 0.5 (Reinaldo et al., 1978). This is a limiting factor when compared with the GAB model which predicts EMCs of water activity from 0.00 to 0.90 (Rizvi, 1995). Similar limitation was also noted for Oswin and Henderson models.

The GAB constant K is a measure of the interactions between the molecules in multilayers with the adsorbent, and tends to fall between the energy value of the molecules in the monolayer and that of liquid water. Lower value of K indicates a much less structured state of the sorbate in the layer following the monolayer, which is the sorbates's pure liquid state. Timmermann et al. (2001) noted that K is practically without exception, near to but less than a unit (1). If it is equal to 1, the multilayer has properties of bulk water, and the sorption behavior could be modeled by the BET equation (Pérez‐Alonso et al., 2006). In this study, the values of K were less than one and indicated that the multilayer has the properties between those of monolayer and the bulk water, which is in agreement with the GAB model assumption. It also means that GAB model could be applied on the leaves irrespective of the drying method used.

The GAB constant C estimated from equation 5 is related to the magnitude of differences between the chemical potentials of monolayer water molecule and multilayer water molecule (McMinn & Magee, 1997). It describes adsorbent (food product)‐adsorbate (water) interactions. As identified by Blahovec and Vanniotis (2008) when C is greater than 2, the GAB model should yield a sigmoid shape curve with point of inflection. If C is less than 2; but greater than 0, the isotherm should be of type III only (isotherm without point of inflection). The C values recorded in the study were greater than 2 for all temperatures studied. This supported the predictions mentioned earlier that isotherm curves obtained were sigmoid. The GAB plots produced negative intercepts and hence, negative values of *C* at some conditions of the samples. Farahnaky et al. (2009) and others reported similar values. The coefficient of determination (r^2^) of GAB model for *Gongronema* leaves ranged from 0.882 to 0.956. Also, correlation coefficient, r, values indicated highly significant (*p* < .05) positive relationship in the predictions using the model. The BET constant C for the adsorption of *Gongronema* leaves was similar to the GAB model.

The BET monolayer moisture content was used to calculate apparent/specific surface area of sorption (So) of Equation 17. The S_o_ values for *Gongronema* leaves as presented in Table 2 ranged from 179.2545 to 249.1489 m^2^/g solid. Oven‐ and sun‐dried samples had higher values than solar‐dried sample. The So values in this study were within the acceptable range of 100–250 m^2^/g solid reported by Tunç and Duman (2007). Labuza (1984) also recorded the same range for most foods. The S_o_ decreased inversely with temperature from 30 to 50°C among the samples. Apparent surface area of sorption is used to determine the water‐binding properties of foods. Previous researches discovered that water adsorption could be affected by surface area, porosity, composition, and the number of binding sites (Yogendrarajah et al., 2015). The amount of water adsorbed by a food material is a function of the affinity between the surface and the water molecules, water vapor concentration, temperature, and the absolute amount of exposed surface area, larger surface area means greater adsorption capacity which gives rise to faster deterioration of food. Solar‐dried leaves adsorbed less water within the range than sun and oven at 30 to 40°C. This has helped to buttress the relationship between BET and GAB models in terms of theoretical background.

Consequently, temperature has a lot of influences on the properties of bound water and surface area of the food product (Sawhney et al., 2011). Values obtained (Table 2) indicated that for the leaf grits, the S_o_ decreased as the temperature increased from 30 to 40°C. Hence, equation 17 showed that S_o_ is directly related to M_o_ which also decreased as the temperature increased within the range as explained above. The results of S_o_ have helped to underscore the moisture adsorption behavior of the leaf grits. Oswin and Henderson models also had better description of the adsorption data based on r^2^ (coefficient of determination for best fit) values that were closer to 1, in addition to the values of r, but versatility in modeling sorption data for agro‐products such as fruits and vegetables, range of water activity required, among others, are limiting factors to relying in their models fully.

In statistical parameters, r^2^ (coefficient of determination of goodness of fit) for all the models were closer to one. However, the RMSE values were less than 2.0 as shown in Table 2 based on GAB and BET models than others. The only exception was oven drying for BET model at 50°C. The %P also followed similar trend where all values calculated using GAB and BET models were less than 10%; than in other models. Nevertheless, BET model had reduced values of χ^2^ for *Gongronema* leaves than in other models as the temperature increased between 30 and 40°C. The GAB model was better in describing the experimental adsorption isotherms data for dried *Gongronema* leaves than other models. This was followed by BET and Oswin models. Henderson model gave the poorest fit to the experimental data among others.

The GAB model from previous studies has been used due to its description of the sorption behavior in a wide range of 0.0–0.93 water activity as shown on this study report. Hence, the model has been used to fit data for more than 50% fruits, vegetables, and meat, especially where moisture adsorption required higher relative humidity range (Andrade et al., 2011). However, some other advantages for using GAB model can be outlined as follows (deuced from other studies): (i) It explains physical adsorption better than others since it was based on the theories of BET and Langmuir; (ii). Its parameters such as C, K, and M_o_; have physical interpretation as it involves sorption processes; which helped to describe temperature effect on isotherms using Arrhenius‐type equation. The GAB model has been successfully applied to sorption studies of potato (Al‐Muhtaseb et al., 2002) and *Gnetum africanum* leaves (Edoun et al., 2010); among others.

### 3.4 Energy models based on thermodynamic properties for *Gongronema* leaf grits

#### 3.4.1 Net isosteric heat of adsorption (differential enthalpy)

**FIGURE 7:**
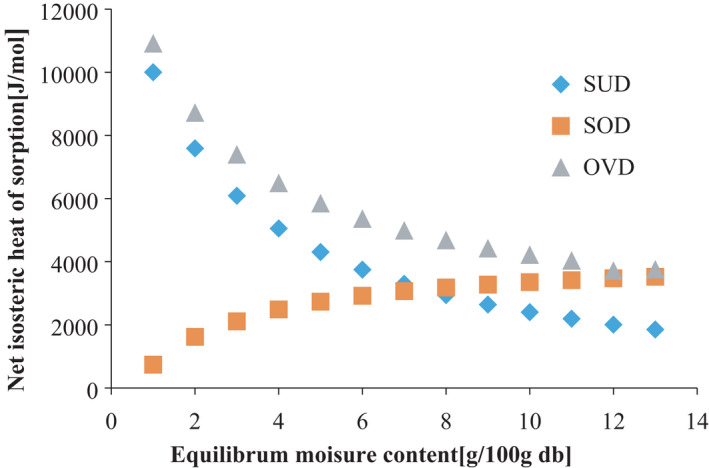
Isosteric heat of moisture adsorption versus equilibrium moisture content of *Gongronema* leaf grits. SUD, sun *Gongronema* leaf grits, SOD, solar *Gongronema* leaf grits and OVD, oven *Gongronema* leaf grits

Differential enthalpy (ΔH‐qst) for the leaf grits is shown in Figure 7. The heat of adsorption varied inversely with EMC of the sun and oven leaf grits but direct variation was found with the solar sample. This inverse variation supported the studies of Samapundo et al. (2007) that verified this parameter for whole yellow dent corn and could be due to highly active polar sites existent on the surface of the food material that were covered with water molecules to form a monomolecular layer. It shows greater water–solid interactions and high binding energy in the form of heat of sorption. Hence, there were strong interactions between water and the leafy powdered compounds that hastened removal of water. As the moisture content increased, the hydrophilic sites became less available, and gave rise to less covering of active sorption site and the formation of a multilayer. This might require less binding energy for removal of water and consequently, less heat of sorption as shown by findings of Pérez‐Alonso et al. (2006) that verified thermodynamic analysis of sorption isotherms of pure and blended carbohydrate polymers.

Hence, the higher heat of adsorption at lower moisture content could be due to greater reluctance by water molecules in migrating from interior to the outer surface of the food product as noted by Ankita and Prasad (2016). However, the direct variation found between EMC and isosteric heat of adsorption could arise from effect of the solar drying environment on the leaves and probably the chemical composition of the leaf powder. This would have caused a very small decrease in isosteric heat of adsorption as temperature increased. The entropy continues to increase from the slight decrease giving rise to less spontaneity of the adsorption process and low moisture binding on the food. The results of net isosteric heat of adsorption assist in providing useful data for energy consumption calculations in the designing of drying equipment, storage process, and understanding of degree of water–solid versus water–water interactions of the leaves.

#### 3.4.2 Differential entropy of adsorption for the leaf grits

**FIGURE 8:**
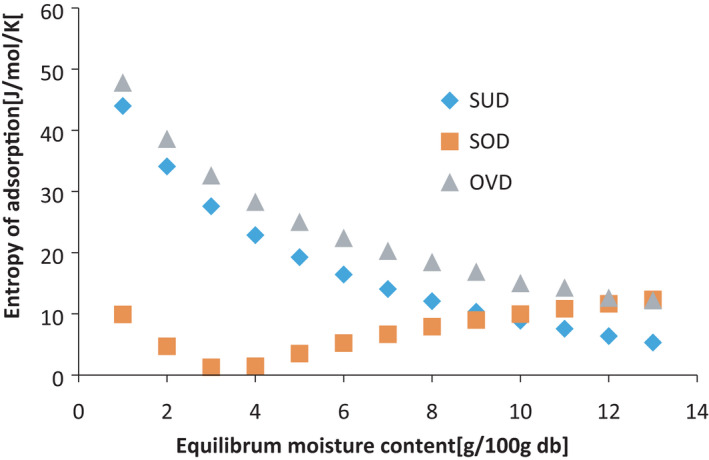
Differential entropy of moisture adsorption versus equilibrium moisture content for Gongronema leaf grits. SUD, sun *Gongronema* leaf grits, SOD, solar *Gongronema* leaf grits and OVD, oven *Gongronema* leaf grits

Differential entropy of moisture adsorption of the *Gongronema* leaf grits is shown in Figure 8. Differential entropy varied inversely with the EMC, despite any of the drying method used. This is because at low moisture content, water adsorbs in the most accessible location on the external surface of the solid. As the moisture content increases the polymer swells, thereby opening up new higher energy sites for water‐binding. Differential entropy of a food material has to do with the number of available sorption sites at a particular energy level and could involve attraction‐repulsive forces in the adsorption system. It is the degree of order or randomness existing in the water‐sorbent system (Mc Minn et al., 2005) and can assist in explaining processes such as dissolution, crystallization, and swelling in the system as suggested by Aviara et al. (2002). The water molecules become more mobile with higher degree of freedom resulting in higher entropy of adsorption. At low moisture content however, the water molecules are tightly bound or ordered by physical and chemisorption forces leading to a loss of degree of freedom and low entropy of adsorption (Ariahu et al., 2006).

#### 3.4.3 Suggested procedure for estimation of shelf life for the leaf grits

Shelf life can represent a time between the production/packaging of the food and that when it becomes unacceptable under certain environmental condition. Out of all the methods for determination of shelf life of a product available, accelerated shelf‐life simulation test was ear‐marked for products that are temperature‐dependent during storage (Phimolsiripol & Seppuku, 2016). The leaf grit products belong to highly perishable foods and are very short shelf life products that can be easily spoiled by enzymatic and microbial reactions once water had been adsorbed/absorbed. Therefore, kinetic reaction approach that involved simulation is proposed for the different products after packaging using modified atmospheric air method.

## 4 CONCLUSION

Moisture adsorption isotherms of *Gongronema* leaf grits showed that sun and oven dried samples adsorbed more moisture than solar dried samples and the isotherm curves for the leaf grits had sigmoid shape. EMC increased directly with water activity at constant temperature. Monolayer moisture content (M_o_) decreased with increase in temperature from 30 to 40°C and subsequently increased at 50°C. The GAB model described the adsorption data of the leaf grits better than other models. Based on the GAB model, the safest M_o_ range for storing *Gongronema* leaf grits would be 7.1036–8.0164 gH_2_O/100 g solid below 40°C.

Differential enthalpy of adsorption decreased with increasing moisture content for the oven sample grits, while moisture adsorption was less spontaneous for the solar leaf grits. Entropy of adsorption decreased with increasing EMC for all the leaf grits. There was increase in concentration of nutrients in the dried leaves than in fresh sample. Hence, the grits when properly packaged could be used as spice or tea powder to manage household nutrient security especially among the elderly in addition to the use as therapeutic foods. Leaf grits could be stored between 30 and 40°C for stable shelf‐life and nutrient retention within 10 and 50% relative humidity at the chosen M_o_. Shelf life of the different leaf products could be determined using accelerated shelf life studies based on Arrhenius chemical kinetic model.

## CONFLICT OF INTEREST

The Authors**:** Ojike, O., *Uzodinma, E.O., Ali, E.O and Nweze, B.C; declare no conflict of interest with any person, Journal, or financial organization.

## AUTHOR CONTRIBUTIONS

**Chigozie F. Okoyeuzu**: Involved in writing—review and editing (supporting); validation (supporting); software (supporting). **Chinwendu R. Eze**: Involved in visualization (supporting); editing (supporting); writing original draft (supporting).

## Data Availability

Data openly available in a public repository that issues datasets with DOIs.
